# Divalent heavy metals and uranyl cations incorporated in calcite change its dissolution process

**DOI:** 10.1038/s41598-020-73555-6

**Published:** 2020-10-08

**Authors:** Xiaohang Zhang, Jianan Guo, Shijun Wu, Fanrong Chen, Yongqiang Yang

**Affiliations:** 1 CAS Key Laboratory of Mineralogy and Metallogeny/Guangdong Provincial Key Laboratory of Mineral Physics and Materials, Guangzhou Institute of Geochemistry, Chinese Academy of Sciences, 511 Kehua Street, Guangzhou, 510640 China; 2 University of Chinese Academy of Science, 19 Yuquan Road, Beijing, 100049 China

**Keywords:** Biogeochemistry, Environmental sciences, Solid Earth sciences

## Abstract

Due to the high capacity of impurities in its structure, calcite is regarded as one of the most attractive minerals to trap heavy metals (HMs) and radionuclides via substitution during coprecipitation/crystal growth. As a high-reactivity mineral, calcite may release HMs via dissolution. However, the influence of the incorporated HMs and radionuclides in calcite on its dissolution is unclear. Herein, we reported the dissolution behavior of the synthesized calcite incorporated with cadmium (Cd), cobalt (Co), nickel (Ni), zinc (Zn), and uranium (U). Our findings indicated that the HMs and U in calcite could significantly change the dissolution process of calcite. The results demonstrated that the incorporated HMs and U had both inhibiting and enhancing effects on the solubility of calcite, depending on the type of metals and their content. Furthermore, secondary minerals such as smithsonite (ZnCO_3_), Co-poor aragonite, and U-rich calcite precipitated during dissolution. Thus, the incorporation of metals into calcite can control the behavior of HMs/uranium, calcite, and even carbon dioxide.

## Introduction

Calcite, the most stable polymorph of CaCO_3_, is the most important and also the most abundant carbonate mineral on Earth^1–3^. The precipitation of calcite serves as a sink of metals^4–7^, organic material^8^, and carbon dioxide^9,10^. Traditionally, lime materials (including calcite, burnt lime, and dolomite) are used to neutralize acidic soils and to overcome the problems associated with soil acidification^11,12^. With the application of calcite, heavy metals (HMs) usually become less bioavailable due to the increase in soil pH and formation of metal–carbonate bounded complexes^13,14^. Furthermore, in situ microbial induced calcite precipitation (MICP) was proposed to remediate soil and underground water contaminated by HMs or radionuclides via substitution/coprecipitation^15,16^. Nevertheless, as much as 30% calcite dissolution was observed, which challenges the long-term sustainability of the calcite formed by MICP^17^.

As a base mineral, the dissolution of calcite can neutralize the acidification of soil and water. During the last two centuries, acidification of Earth's air, water, and soil has been accelerated due to anthropogenic activities, such as the combustion of fossil fuels and smelting of ores, mining of coal and metal ores, and application of nitrogen fertilizer to soils^18,19^. Due to ocean acidification^20^, the dissolution of marine CaCO_3_ (including sediments and coral reef) has been reported worldwide^21–24^. On the continent, the concentrations of Ca in freshwater increased due to terrestrial rock dissolution as a result of climate change and anthropogenic acid deposition^25,26^. Meanwhile, the carbonate bonded metal will release into the environment, which could make calcite a potential source of heavy metals^27,28^. For example, uranium concentrations in river water are primarily determined by the dissolution of limestone (dominated by calcite)^29^. Strontium (Sr) released from Himalayan carbonate changed the Sr isotope composition in seawater and marine limestones^30^. In the Karst area, the weathering of carbonate rock naturally causes HMs to accumulate in soil^31–34^, resulting in HM pollution in plants^35^.

The growth and dissolution of calcite have been investigated extensively and reviewed in documents^36–38^. Generally speaking, the dissolution of calcite is influenced by the temperature, pH, ${\mathrm{P}}_{{CO}_{2}}$, solution composition and inhibitors^36–39^. However, the influence of impurities in calcite on its dissolution is not well understood. Based on atomic force microscopy (AFM) observations, Harstad and Stipp concluded that Fe^2+^, Mg^2+^, Mn^2+^, and Sr^2+^, which are naturally present in Iceland spar calcites, inhibited the dissolution of calcite^40^. However, at least for Mg^2+^ and Mn^2+^, this conclusion conflicts with the experimental data obtained from magnesian calcite^41–44^ and synthesized Mn^2+^ containing calcite^45^. The macroscopic dissolution experiment of natural inorganic, biogenic, and synthesized samples demonstrated that the dissolution ability of magnesian calcite is positively correlated with the content of Mg in calcite^41–43^, which is supported by the AFM observation, according to Davis and his coauthors^44^. Therefore, high-Mg calcite in tropical continental shelf sediments is more sensitive than low-Mg calcite to ocean acidification^46^. Recently, we found that the incorporation of Cu^2+^ and Mn^2+^ enhanced the solubility of calcite^45^. The above-mentioned studies indicated that the influences of impurities on calcite dissolution is complex and need further investigation.

Because of the multiformity of heavy metal contamination in the field and the numerous impurities in natural calcite, it is difficult to identify the influence of a single component on its dissolution. Herein, we provide further evidence on the dissolution behavior of synthesized calcite incorporated with Cd^2+^, Co^2+^, Ni^2+^, Zn^2+^ and ${\mathrm{UO}}_{2}^{2+}$, which are common environmental pollutants. The results showed that the solubility of calcite was inhibited by coprecipitated Cd^2+^ and Ni^2+^ but enhanced by Co^2+^. Unexpectedly, Zn^2+^ and ${\mathrm{UO}}_{2}^{2+}$ showed both inhibiting and enhancing effects, depending on the mass of the impurities in calcite. These observations suggested that the incorporated HMs and radionuclides might control the dissolution of calcite. Inversely, the migration of HMs and radionuclides could be remarkably controlled by the host minerals as well.

## Results

### Characterization of calcite incorporated with impurity metals

With the addition of HMs, the XRD patterns of all the Cd and Zn containing products were the same as the pure calcite reference pattern, indicating that no detectable secondary crystalline phases were present (Fig. S1a,b, Table 1). However, traces of aragonite were found in sample Co-10 (Fig. S1c), while vaterite was present in U-08 and U-10 (Fig. S1e). Meanwhile, both aragonite and vaterite occurred in Ni-0.4 and Ni-01 (Fig. S1d). To avoid the impact of aragonite and vaterite, we used samples without detectable secondary phases for further experiments.

**Table 1:** Mineral/chemical compositions and BET specific surface areas of the synthesized samples.

Sample	Mineral composition	M^2+^/Ca^2+^ (mol%)		BET specific surface area (m^2^/g)
		Added	Measured	
M0	Cal*	–	–	0.17
Cd-02	Cal	2.00	1.83	0.38
Cd-04	Cal	4.00	4.05	0.57
Cd-06	Cal	6.00	6.75	1.02
Cd-08	Cal	8.00	9.31	1.57
Cd-10	Cal	10.00	11.73	1.66
Zn-02	Cal	2.00	1.89	0.43
Zn-04	Cal	4.00	3.18	1.65
Zn-06	Cal	6.00	4.83	1.69
Zn-08	Cal	8.00	7.04	2.93
Zn-10	Cal	10.00	12.36	4.49
Co-02	Cal	2.00	1.37	0.25
Co-04	Cal	4.00	3.43	0.53
Co-06	Cal	6.00	6.40	0.80
Co-08	Cal	8.00	7.72	1.39
Co-10	Cal, arg*	10.00	–	–
Ni-0.04	Cal	0.04	0.05	0.19
Ni-0.08	Cal	0.08	0.07	0.21
Ni-0.2	Cal	0.20	0.28	0.22
Ni-0.4	Cal, vat, arg	0.40	–	–
U-0.5	Cal	0.50	0.56	0.51
U-01	Cal	1.00	0.95	0.69
U-02	Cal	2.00	1.55	1.46
U-04	Cal	4.00	2.64	4.64
U-08	Cal, vat*	8.00	–	–

*Cal, vat, and arg are abbreviations for calcite, vaterite, and aragonite, respectively.

**Figure 1:**
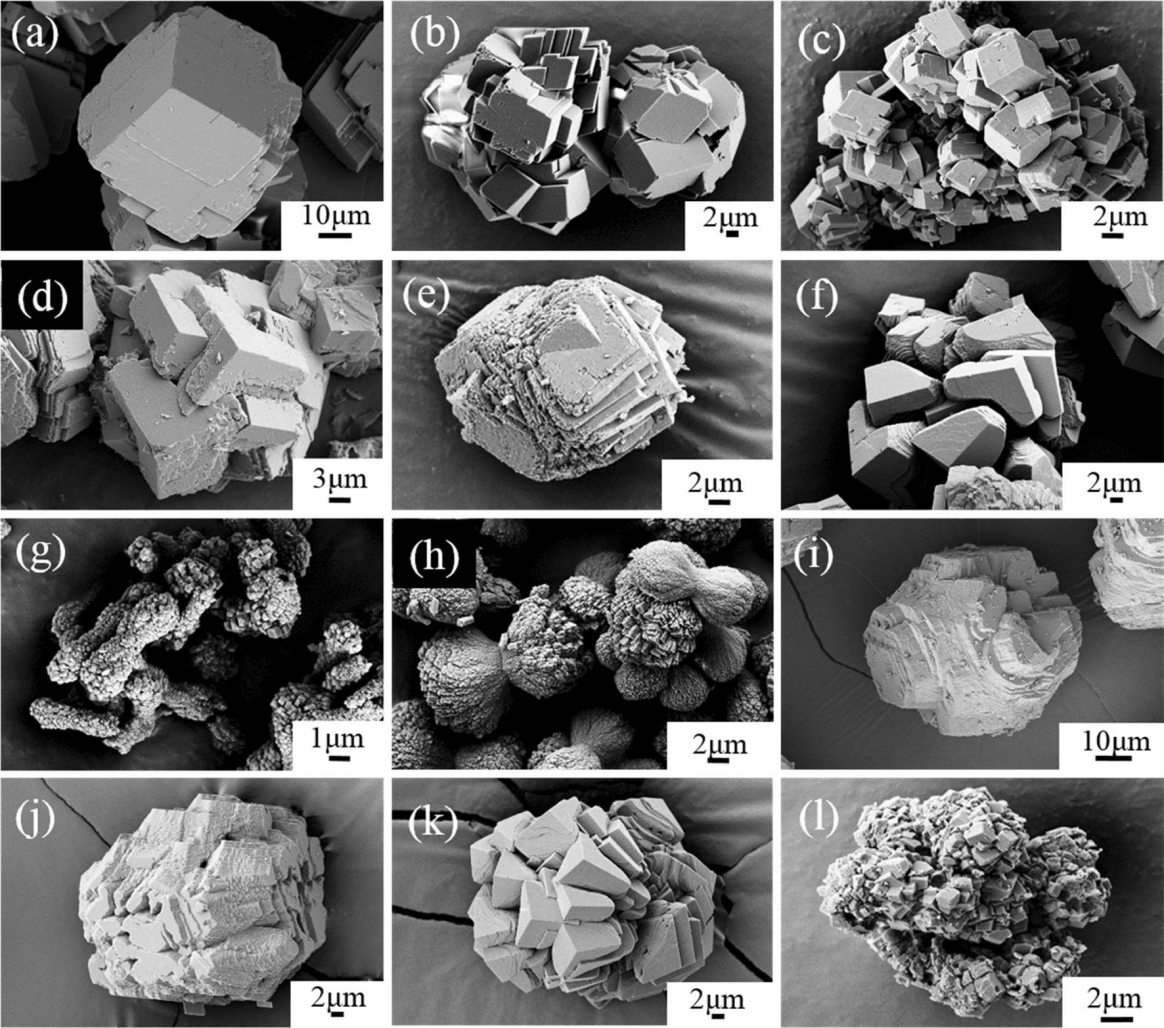
SEM images of selected calcite. (**a**) M0, (**b**) Cd-02, (**c**) Cd-10, (**d**) Zn-02, (**e**) Zn-10, (**f**) Co-02, (**g**) Co-06, (**h**) Co-08, (**i**) Ni-0.04, (**j**) Ni-0.2, (**k**) U-0.5, and (**l**) U-04.

As shown in Fig. 1a, pure calcite showed a typical rhombohedral morphology as a euhedral calcite crystal. With the incorporation of metals, the morphology changed to aggregates of semi-euhedral or anhedral (dumbbell) phases with small sizes (Fig. 1b–l). This trend is supported by the BET surface area data, which are positively correlated with the molar fraction of metals, with R^2^ values of 0.65 (Ni), 0.89 (Co), 0.93 (U), and 0.96 (Cd, Zn) (Table 1, Fig. S2). Usually, the incorporation of impurities will decrease the sizes of crystals^47,48^ due to the inhibition of the crystal growth.

Figure S3 showed the spatial distribution of metals in typical polished HM-calcite. Both of the line scan and elemental mapping results showed the presence of Cd/Zn-rich cores in Cd/Zn-calcite (Fig. S3a–h). However, the Co-calcite and Ni-calcite were quite homogenous in general. Interestingly, U-calcite possessed two distributions, as mentioned above, i.e., a U-rich core and homogenous distribution.

### Release of metals during the dissolution of calcite

**Figure 2:**
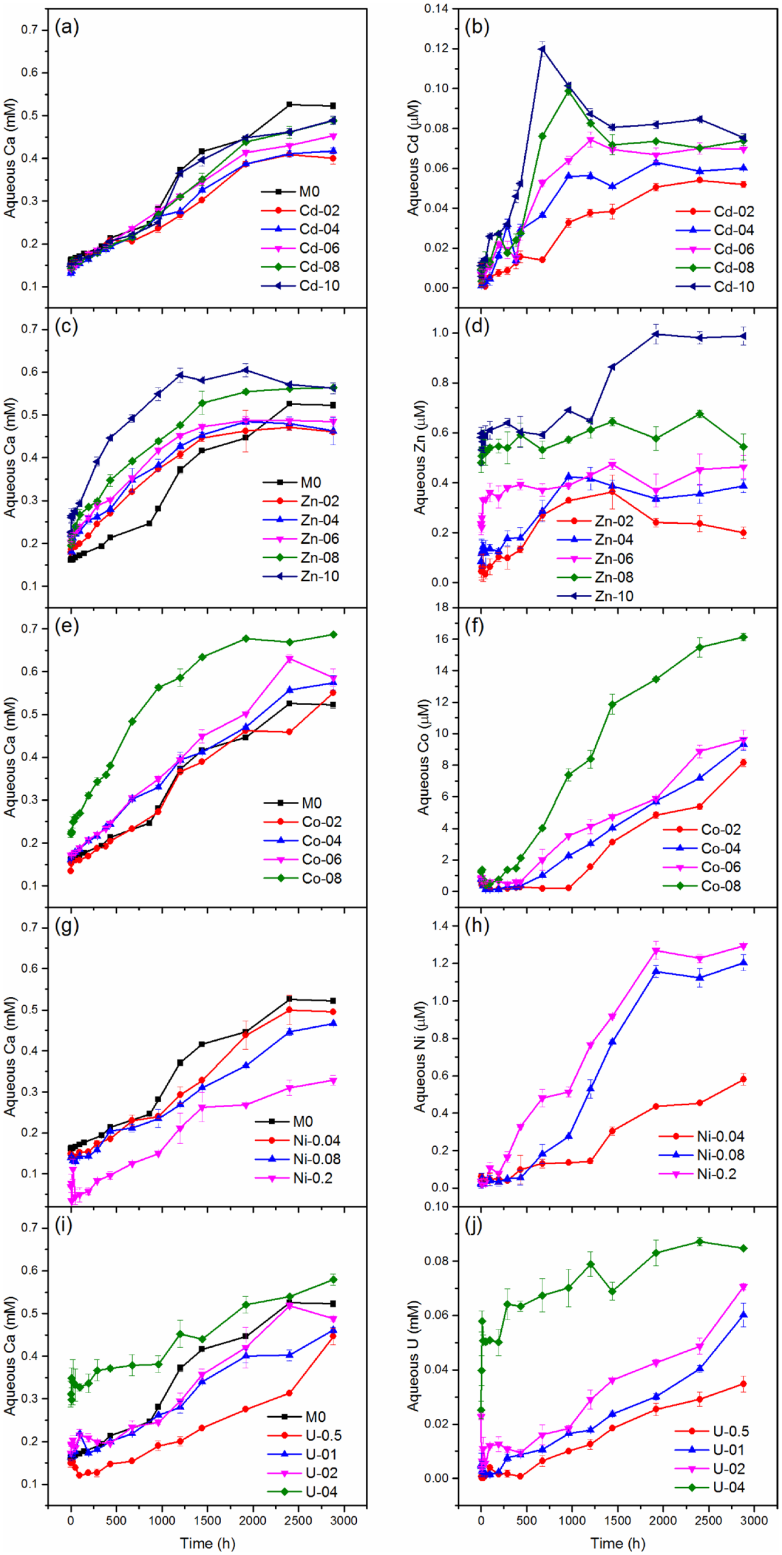
Evolution of the aqueous metal concentration during the dissolution of calcite. Left panels, the concentration of dissolved Ca^2+^. Right panels, the concentration of dissolved HMs, and uranium.

Once calcite contacted the solution, dissolution occurred immediately, especially in an acidic solution. For example, when the initial solution pH ranged from 1.0 to 8.9, the proportion of pure calcite that dissolved within 20 min accounted for 83.8–97.4% of the total mass dissolved within 120 min (Fig. S4). However, the dissolution process proceeded until equilibrium was reached at approximately 2400 h with an initial pH of 5.0. (Fig. 2). The total dissolved calcium and solution pH at equilibrium for pure calcite were 0.52 mM and 8.19 (Figs. 2, S5), respectively, which are both very close to the theoretical data (0.53 mM and 8.22) in an open system. There was 5 mL of air in each of the closed tubes in our experiment, resulting in a semi-open system. Since the annual global average carbon dioxide (CO_2_) concentration on Earth's surface was 407.4 ± 0.1 mg L^−1^ in the year 2018^49^, the closed CO_2_ in each tube was no less than 0.046 mmol, which can dissolve into the alkaline solution. However, this tiny CO_2_ amount was not enough to increase the DIC up to 1.03 mmol at equilibrium. Therefore, the effect of environmental CO_2_ on the experiment was not significant.

With the incorporation of HMs and uranium, the quantity of dissolved calcium changed, even at the early stage (first two hours) of dissolution (Table S2). After two hours of dissolution, the dissolved Ca concentrations were 0.13–0.15 mM and 0.15–0.07 mM for all the Cd- and Ni-incorporated calcite (Cd/Ni-calcite), which were less than the dissolved Ca concentration in the control (0.16 mM) (Fig. 2a,g and Table S2). On the contrary, more Ca was dissolved in Zn-calcite than in Cd/Ni-calcite (0.18–0.23 mM) (Fig. 2c and Table S2). However, Co and U showed inhibition effects at low contents (0.14 and 0.15 mM for Co-2 and U-0.5) and an enhancement at a high content (0.17–0.22 mM for Co-06/08 and 0.16–0.31 mM for U-01/02/04) (Fig. 2e,i and Table S2). When calibrated with the BET surface area, the dissolution rates within the first 2 h for all heavy metal-incorporated calcites (2.90–31.70 μmol/m^2^·h) were less than those for the pure calcite (37.36 μmol/m^2^·h) (Fig. S6). This suggested that the dissolution rate was controlled both by the mineral surface area and the fraction of HMs. After 2880 h, less Ca dissolved for Cd-calcite (0.40–0.49 mM) and Ni-calcite (0.50–0.33 mM) than for pure calcite (0.52 mM) (Fig. 2a,g), while more Ca dissolved for Co-calcite (0.55–0.69 mM) (Fig. 2e) than for pure calcite. Surprisingly, Zn and U showed both inhibition (0.46–0.48 mM for Zn-02/04/06 and 0.45–0.49 mM for U-0.5/01/02) and enhancement effects (0.56 mM for Zn-08/10 and 0.58 mM for U-04), depending on the proportion of impurities in calcite (Fig. 2c,i).

As expected, all the HMs and uranium released into the solution together with calcium, and the amounts released increased with time until reaching equilibrium, excepted for Co-calcite, Ni-0.04, and U-0.5/01/02, which did not reach equilibrium, even after 2880 h (Fig. 2f,h,j). Note that Cd-08 and Cd-10 displayed peak values of Cd at 960 h and 672 h (Fig. 2b), which is similar to the phenomenon observed for Cu-calcite and Mn-calcite^45^. The final released HM concentrations were 0.05–0.08 μM for Cd (Fig. 2b), 0.20–0.99 μM for Zn (Fig. 2d), 8.15–16.13 μM for Co (Fig. 2f), 0.58–1.29 μM for Ni (Fig. 2h) and 34.78–84.76 μM for U (Fig. 2j). The proportions of released Cd, Zn, Co, Ni and U were 0.01–0.02‰, 0.06–0.09‰, 1.11–4.03‰, 0.31–1.22‰ and 3.29–9.96% of the total loaded metals, respectively.

As demonstrated in Fig. S7, the molar ratios of total dissolved metals were 0.09–0.18‰ for Cd/Ca, 0.43–1.75‰ for Zn/Ca and 1.48–2.35% for Co/Ca, which were much less than those in the solids, except for Co-02, suggesting the nonstoichiometric release of Cd, Zn, Co and Ca. Contrarily, the molar ratios of released Ni/Ca and U/Ca were 1.17–3.93‰ and 7.23–14.63%, which are both higher than those in the solids (0.5–2.8‰ and 0.56–2.64%). These data demonstrated that all the studied HMs and U showed nonstoichiometric release behaviors during the dissolution of calcite. In particular, Ni and U tend to passively release into the solution, while Cd, Zn, and Co prefer to prevent dissolution.

**Figure 3:**
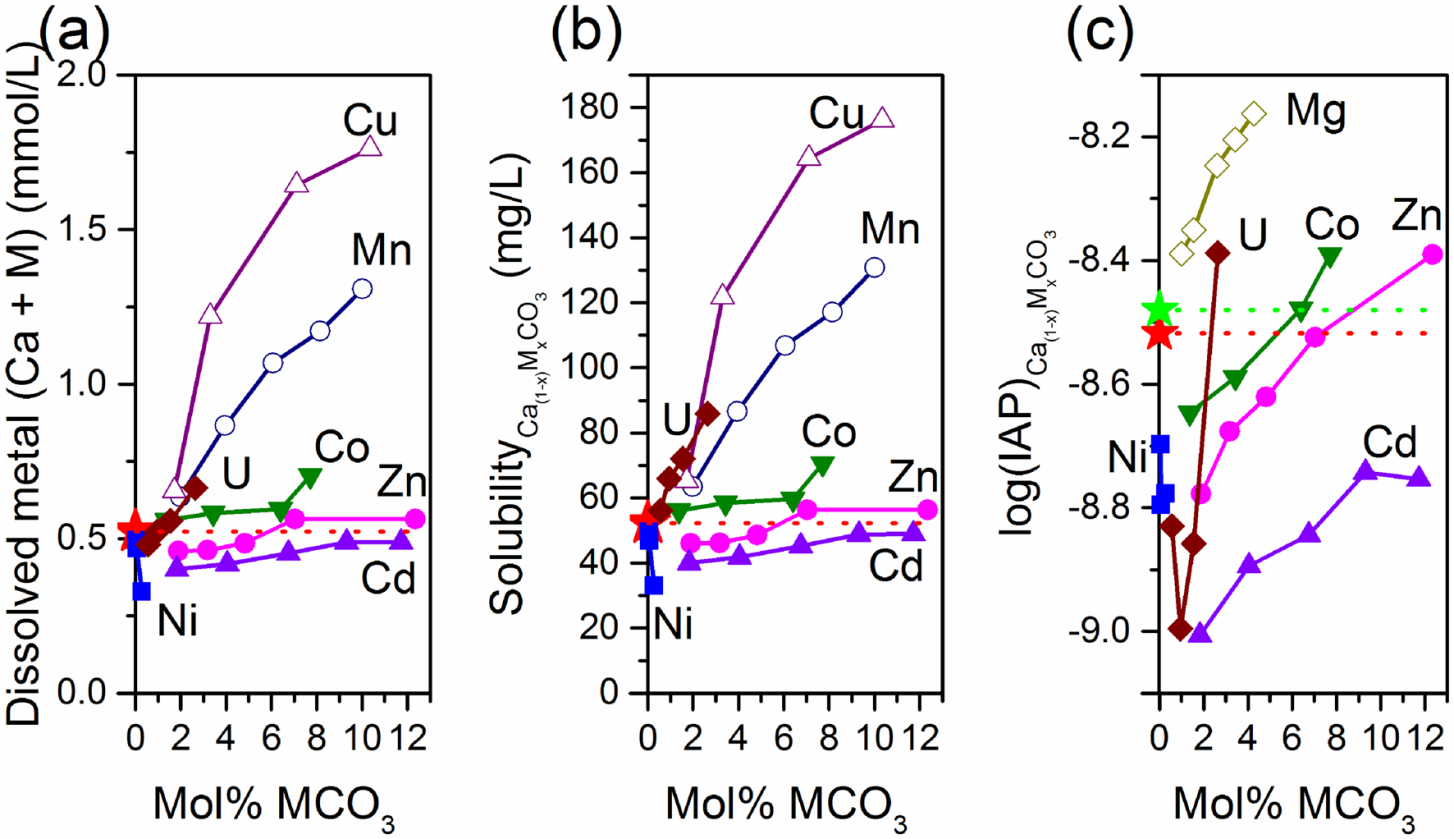
The concentration of total dissolved metals, the calculated solubility, and log(IAP) varied with the content of HMs. The red and green star/dashed lines indicate the result of the pure calcite obtained in this investigation and from the Minteq(V4) database in Phreeqc. $\mathrm{IAP}={\mathrm{\alpha }}_{{\mathrm{Ca}}^{2+}}\bullet {\mathrm{\alpha }}_{{\mathrm{CO}}_{3}^{2-}}$. The data of Cu and Mn were taken from Zhang et al.^45^, and the data of Mg were obtained from Davis et al.^44^.

Figure 3 showed the total dissolved metals (Ca + M) and the corresponding solubility at 2880 h. Since the content of impurities is low in calcite, the addition of released HMs did not change the relationship between the total dissolved metals and the content of impurities (Fig. 3a). The calculated solubility was 52.26 mg/L for pure calcite, 40.03–48.90 mg/L for Cd-calcite, and 49.59–32.99 mg/L for Ni-calcite, showing the inhibition of calcite dissolution with the incorporation of Cd and Ni. Comparably, the solubility of Co-calcite and U-calcite enhanced to 56.05–70.62 mg/L and 56.13–85.90 mg/L, respectively. Meanwhile, the solubility of Zn-calcite showed both inhibition (46.05–48.54 mg/L for Zn-02/04/06) and enhancement (56.41–56.37 mg/L for Zn-08/10) (Fig. 3b). As is known, the solubility of a material at equilibrium is independent of its surface area. Therefore, the relationship between the solubility of calcite and the molar fraction of HMs was fitted using linear equations, which provided an R^2^ value of 0.96, 0.79, 0.69, 0.99 and 0.98 for Cd, Zn, Co, Ni, and U, respectively (Table S3). Except for Ni, the solubility of calcite is positively correlated with the molar fractions of HMs, which are the same as those of Mg-calcite^41–44^, Cu-calcite, and Mn-calcite^45^.

K_sp_ and IAP are generally used to characterize the solubility of a material and are calculated using the ion concentration or activity. As shown in Fig. 3c, the data of all the IAP values were calculated using the ion activity of Ca^2+^ and ${\mathrm{CO}}_{3}^{2-}$ according to Eq. (8). In addition to U, the graphs of Cd, Zn, Co, and Ni showed a tendency similar to that shown in Fig. 3a and b. When we tried to calculate the IAP using Eq. (9), we obtained results similar to those shown in Fig. 3c. Since the ion activity cannot be measured directly, we recommend using the total concentration of metals to express the solubility of impure calcite. This method should be more convenient in the field.

### Morphology change and precipitation of secondary minerals during the dissolution of calcite

**Figure 4:**
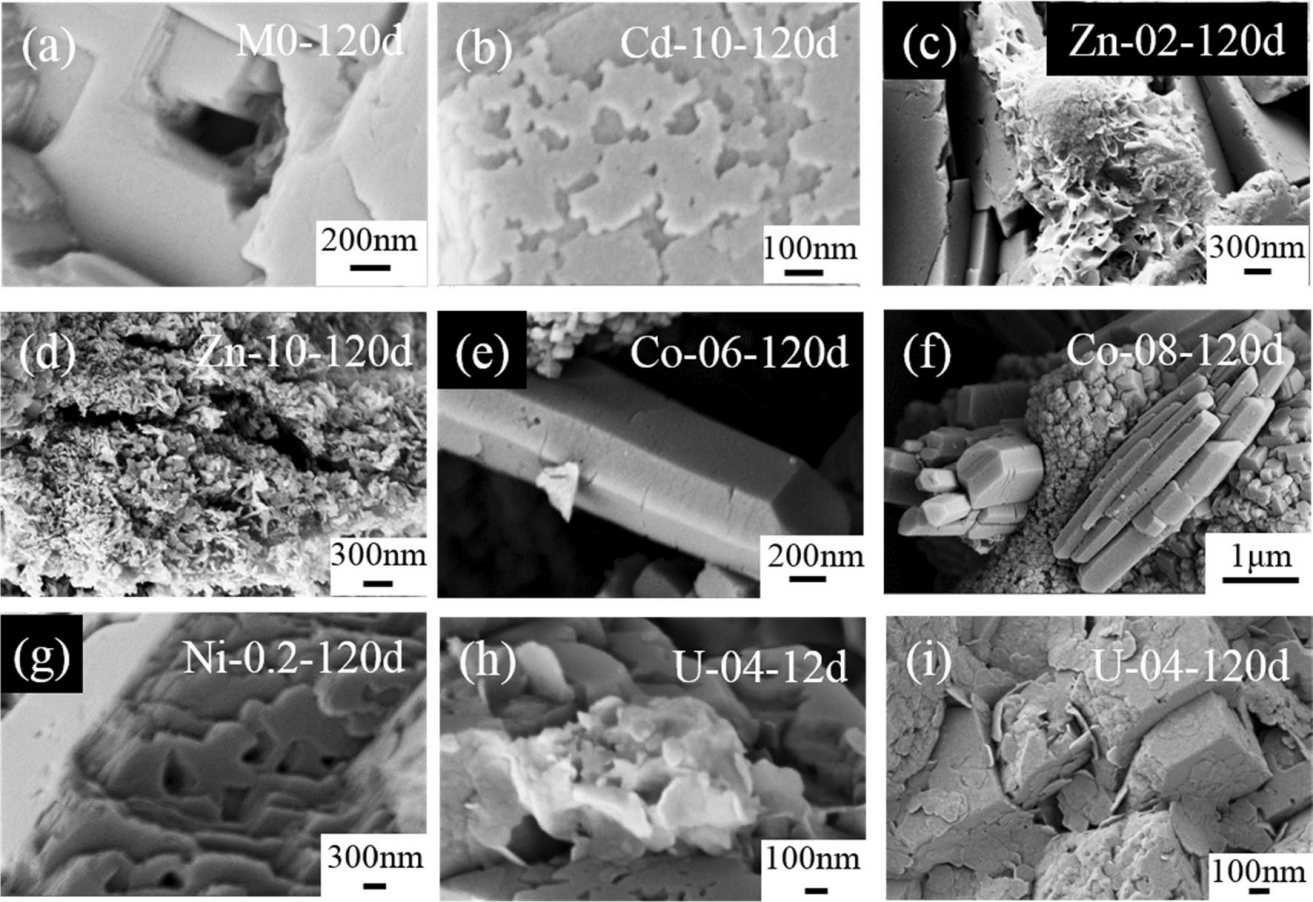
SEM images of selected calcite after dissolution. Sample names on the top-right of each figure were labeled as sample name-T, e.g., Cd-10-120d stands for Cd-10 after 120 days of dissolution.

After dissolution, typical etch pits were present on the surfaces of pure calcite (Figs. 4a, S8a–f) and Cd/Ni-calcite (Figs. 4b,g, S8g–l). Unexpectedly, some secondary minerals precipitated on the surface of Zn/Co/U-calcite (Figs. 4c–f,h,i, S9, S10). These secondary minerals were composed of lamelliform nanocrystals for Zn/U-calcite and prism nanocrystals for Co-calcite. Meanwhile, the secondary minerals formed earlier in the samples with a high content of impurities than in the one with a low content, i.e., new precipitates were first observed after 12 and 28 days for U-04 and U-02, respectively. As shown in Table 2 and Fig. S11, the EDS data demonstrated that the secondary minerals of Zn-10 and U-04 were rich in Zn and U, while Co-08 contained trace Co, which was distinguished with the calcite before dissolution.

**Table 2:** Chemical molar compositions of the secondary minerals obtained by TEM-EDS.

Sample	Secondary minerals			Calcite before dissolution
	Ca%	M%	(M/Ca)%	(M/Ca)%
Zn-10	0.08	22.14	27.675	12.36
Co-08	23.82	0.22	0.92	7.72
U-04	4.09	1.17	28.61	2.64

**Figure 5:**
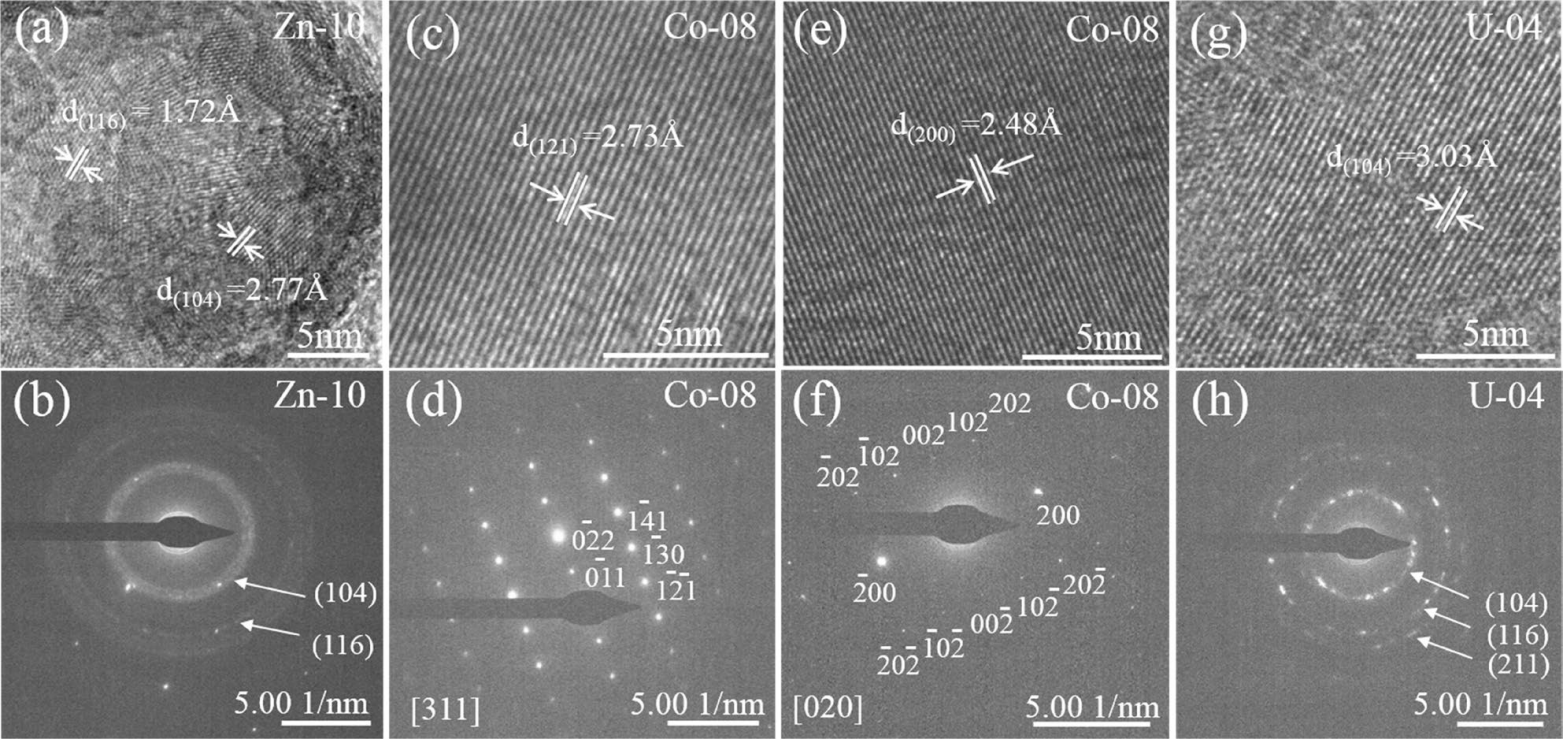
HRTEM images and SAED patterns of selected secondary minerals.

**Figure 6:**
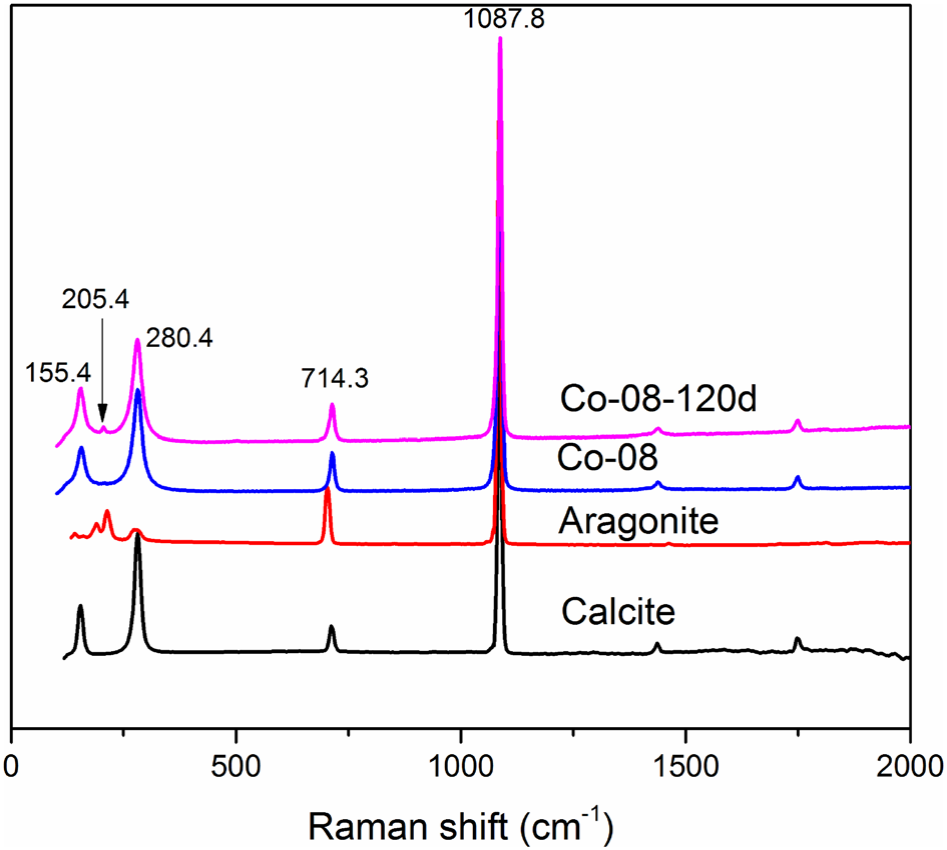
Raman spectra of the secondary mineral of Co-08.

Furthermore, high resolution transmission electron microscopy (HRTEM) and selected area electron diffraction (SAED) were used to characterize the secondary minerals (Fig. 5). The HRTEM image of the secondary minerals of Zn-10 (Fig. 5a) revealed the lattice fringes of the smithsonite (ZnCO_3_) (104) plane (d = 2.77 Å) and (116) plane (d = 1.72 Å) (RRUFF^50^), which was further validated by the SAED patterns (Fig. 5b). This observation agreed with the content of Zn being much higher than that of Ca, according to the EDS data. For the secondary mineral of Co-08, the HRTEM images show lattice spacings of 2.73 Å and 2.48 Å (Fig. 5c,e), which can be indexed to the (121) and (200) planes of aragonite (RRUFF^51^). The relative SAED patterns (Fig. 5d,f) further proved that they are single crystals of aragonite along the [311] and [020] directions. Moreover, the elongated prismatic morphology supported the occurrence of aragonite (Figs. 4e,f, S10c–h). Furthermore, the Raman peak at 205.4 cm^−1^ confirmed that aragonite was a secondary mineral in Co-08-120d (Fig. 6). Comparably, the HRTEM images of the secondary minerals of U-04 possessed lattice fringes with a d-spacing of 3.03 Å, which corresponded to the (104) reflection of calcite (RRUFF^52^) (Fig. 5g), as confirmed by the SEAD pattern (Fig. 5h). Therefore, the secondary minerals observed in our experiment were Ca-poor smithsonite, U-rich calcite, and Co-poor aragonite.

## Discussion

### The incorporation of impurities in calcite

Divalent cations, particularly those with an ionic radius close to that of Ca^2+^, easily substitute for Ca^2+^ in the structure of calcite^53^. The experiments and simulation well demonstrated the incorporation of Cd^2+^^54–62^, Co^2+^^55,57,62–68^, Zn^2+^^58,62,64,65,69–75^, Ni^2+^^76–79^ and ${\mathrm{UO}}_{2}^{2+}$^80–91^ into the structure of calcite. In this study, the presence of aragonite and vaterite indicated the favoritism of these phases in the high concentration Co, Ni, and U system. The incorporated Co, Ni, and U in CaCO_3_ could stabilize aragonite or vaterite phases, agreeing well with previous investigation^66,83^. Taking U as an example, a previous study reported that the U concentration in synthesized aragonite was higher than that in calcite, even though both of them grew in a solution containing the same concentration of uranium^90^. This phenomenon could be attributed to the thermodynamic preference of U incorporation into aragonite^92^. Another reason was that aragonite has a higher surface area than calcite and can absorb more U on its surface. Aragonite was present only in Co-10, even though equal stoichiometric amounts of HMs were used for the synthesis of Cd-10, Zn-10, and Co-10. The exception of Co-10 demonstrated that the tolerance of the calcite structure for the impurity metals would be varied with its ionic radii, following the Goldschmidt’s Rules.

Traditionally, it is believed that the incorporated divalent cations would take the crystallographic site of Ca^2+^^53^. However, it is difficult to determine the coordination environment and, thus, the incorporation modes of the impurities until the development of extended X-ray absorption fine structure spectroscopy (EXAFS). According to the experimental EXAFS data and Ab initio molecular dynamics simulation, there are at least five models of incorporating impurities in calcite. (1) Isovalent substitution occurs for Ca^2+^ with sixfold coordination, which is the same as the coordination of Ca^2+^ in calcite, e.g., Ba^2+^, Cd^2+^, Co^2+^, Mn^2+^, Pb^2+^, Sr^2+^ and Zn^2+^^64,65,69,93–95^. (2) Aliovalent substitution occurs for Ca^2+^ with six-fold coordination, e.g., Nd^3+^, Sm^3+^^96^, Np^5+^^97^, U^4+^^98^ and U^6+^ (as ${\mathrm{UO}}_{2}^{2+}$) in natural calcite^84,87^. (3) Aliovalent substitution occurs for Ca^2+^ with seven-fold coordination, e.g., Dy^3+^, Yb^3+^^96^, Am^3+^^99^ and U^6+^ (as ${\mathrm{UO}}_{2}^{2+}$) in synthesized calcite^80,81^. (4) Impurities can occupy the ${\mathrm{CO}}_{3}^{2-}$ site, e.g., ${\mathrm{IO}}_{3}^{-}$^100,101^${\mathrm{CrO}}_{4}^{2-}, {\mathrm{SeO}}_{3}^{2-}, {\mathrm{AsO}}_{3}^{3-}$^101–104^ and ${\mathrm{AsO}}_{4}^{3-}$^105^. (5) As the second phase or nanophase impurity, e.g., Ba occurs dominantly as BaSO_4_ in natural Ba-rich calcite^64^, nano hydrozincite (Zn_5_(CO_3_)_2_(OH)_6_) and sphalerite (ZnS) discovered in foraminiferal calcite shells^69^. Furthermore, the incorporation of monovalent cations, i.e., Na^+^ and Li^+^, might be required to achieve a local charge balance in the calcite lattice^98,106^. In Zn-contaminated foraminiferal calcite shells, adsorption, isovalent substitution, and second nanophases coexisted with each other^69^. Coexisting multimechanisms are probably much more common in the natural system and high concentrations of impurities than in the other systems.

The spatial distribution of trace elements in biogenic calcite might be homogeneous or heterogeneous, which relates to the temperature, growth rate, or organic matter content^69,107–110^. Due to the overgrowth of crystals, the heterogeneous distribution of impurities has been discovered in natural single crystals of low-temperature precipitated calcite^7,111^. In laboratory-synthesized calcite, the distribution of trace elements is not reported very often, even though heterogeneous distribution sometimes occurrs^66,112^. However, the HM-calcites in our experiment showed both heterogeneous (Cd, Zn, U) and homogeneous (Co, Ni, U) distributions. The homogeneous distribution of Co in Co-08 differed from the result of Katsikopoulos et al.^66^, probably due to the visible difference in the Co fractions in the solid phases, i.e., a Co/Ca molar ratio of 0.077 in Co-08 versus 1.67 in the previously published data. Unexpectedly, some U-04 particles showed heterogeneous distributions of uranium, while other particles showed homogeneous distributions. This phenomenon indicated the complexity of the distribution of impurities in HM-calcite, which required further investigations.

### The influence of incorporated impurities on the dissolution of calcite

Since the natural water chemistry, e.g., seawater chemistry, is complex, numerous investigations have demonstrated that the dissolution of calcite is affected by the water constituents, including the major, minor, and trace constituents, regardless of whether the constituents are inorganic or organic^36–38^. Based on the inhibition dissolution of natural calcite, e.g., limestone and marble, Svensson and Dreybrodt suggested that Ca^2+^ ions are adsorbed to impurities at the surface of the natural mineral to form a complex, which acts as an inhibitor^113^. Subsequently, Eisenlohr et al. suggested that aluminosilicate nano-complexes could incorporate in the calcite matrix. During dissolution, these impurities will release from the calcite matrix and then will be adsorbed irreversibly at the reacting surface, where they act as inhibitors^114^.

Except for Mg^2+^, the influence of the impurities incorporated metals in solids on the dissolution of calcite is unknown. According to the dissolution of magnesian calcite, the Mg^2+^ in calcite enhanced the mineral solubility through magnesium incorporation and inhibited calcite growth^41–44^. On the one hand, high magnesian calcite compositions exist within the spinodal gap and are therefore unstable. On the other hand, calcite growth is inhibited through either step-blocking by Mg^2+^ adsorption and slow dehydration or enhanced the mineral solubility associated with Mg^2+^ incorporation^41–44^. Contrarily, Harstad and Stipp reported that naturally present trace metals (Fe^2+^, Mg^2+^, Mn^2+^, and Sr^2+^) inhibited the dissolution rate of calcite based on AFM observations^40^. Recently, we demonstrated the enhanced dissolution of calcite once Cu^2+^ and Mn^2+^^45^ were incorporated. These results contradicted each other with regards to the role of Mg^2+^ and Mn^2+^ play in calcite dissolution.

Firstly, the calcite used by Harstad and Stipp contained many impurity metals. Therefore, the enhancement effect contributed by Mg^2+^ and Mn^2+^ might be neutralized by other inhibitors, such as Fe^2+^ and Sr^2+^. Secondly, the relationship between the impurity molar fractions was inconsistent with the solubility/dissolution rate, as shown in Figs. 3 and S6. It has been observed that the surface area-normalized dissolution rates increase non-linearly with decreasing the initial crystal size^115^. However, the normalized dissolution rates of our impurity-containing calcite with small crystal sizes were lower than that of the large pure calcite, suggesting the inhibition of the dissolution rate by the impurities. This inconsistency indicates that the impurity-containing calcite required a longer time than pure calcite to reach equilibrium, as described previously. According to Fig. 2, the pure calcite reached equilibrium at approximately 2880 h, while Cd-calcite, Ni-calcite, and U-calcite still have an upward trend, suggesting that Cd/Ni/U-calcite did not reach equilibrium at that time. Third, the dissolution rate obtained from reactor experiments performed on crushed crystals (i.e., mineral powders) differed from that measured locally at the surface of polished or pristine crystals^116^. This phenomenon is ascribed to the differences in the surface reactivity distribution at the two scales of observation and different sample preparation methods, which could change the distribution of reactive sites^116^. In our samples, the BET surface area increased with the increase in the molar fraction of impurities, which partially due to the small crystal size. Compared to large crystals, the crystals with small sizes will provide more crystal edges, which can control the dissolution at the crystal scale. However, the contribution of the edges to the overall dissolution is crystal-size- and time-dependent, which is not well understood yet^116^. Furthermore, one should be very careful to hand these phenomena since the surface area-normalized dissolution rates increase non-linearly with decreasing initial crystal sizes^115^. Last but not least, the heterogeneous distribution of impurities in Cd/Zn/U-calcite should contribute more or less to the nonstoichiometric release of Cd, Zn, and U. We propose that the nonstoichiometric release of Cd, Zn during the weathering of Cd/Zn-bearing limestone is one of the reasons that Cd and Zn enrich the soil in the Karst area^31–34,117^.

### The precipitation of secondary minerals during the dissolution of calcite

In principle, the chemical weathering of rocks involves the coupled dissolution of the primary minerals and precipitation of more stable secondary minerals. The precipitation of secondary minerals is significant for the formation of soil. For example, feldspar, one of the most abundant rock-forming minerals, chemically reacts with water and water-soluble compounds to form clay, such as halloysite and kaolinite^118,119^. In this case, the principal components of the primary mineral (feldspar) (Al and Si) are inherited by the secondary minerals (halloysite and kaolinite).

The trace elements, such as the rare earth elements (REEs), might be enriched or depleted in the altered products depending on the soil and groundwater pH^120^. In South China, the REEs adsorbed on the secondary clay minerals develop world-class heavy rare earth element (HREE) deposits, known as regolith-hosted HREE deposits or ion-adsorption REE deposits, which provide most of the HREE produced in the world^121,122^. Another interesting example is the fate of trace arsenic (As) during the dissolution of As-containing pyrite; As might be adsorbed by the secondary goethite or might be incorporated into the secondary jarosite via substitution for sulfate^123^. Therefore, the minor/trace elements in minerals are hard to form independent secondary minerals during dissolution/weathering. During the dissolution of (Pb_x_Ca_1−x_)_5_(PO_4_)_3_OH in a laboratory, both PbHPO_4_ and Pb_3_(PO_4_)_2_ formed as secondary products when the Pb molar fraction in the solid solution was high enough (x ≥ 0.3)^124^.

During the dissolution of calcite, the precipitation of several secondary minerals has been observed in the presence of aqueous cations (Pb^2+^, Cu^2+^, and ${\mathrm{UO}}_{2}^{2+}$)^125–128^, oxyanions (${\mathrm{PO}}_{4}^{3-}$, ${\mathrm{AsO}}_{4}^{3-}$, ${\mathrm{SbO}}_{4}^{3-}$ and ${\mathrm{SeO}}_{3}^{3-}$)^129,130^, and F^−^^131^ due to the formation of less soluble phases. Sometimes, calcite might be entirely replaced by the secondary minerals, such as cerussite (PbCO_3_)^132^, dolomite (CaMg(CO_3_)_2_)^133–135^, magnesite (MgCO_3_)^134^, siderite (FeCO_3_)^133^, gypsum (CaSO_4_)^136,137^, whewellite (CaC_2_O_4_·H_2_O)^138^ and fluorite (CaF_2_)^139,140^, via a coupled dissolution–precipitation mechanism. Interestingly, the capture of aqueous Mg on the calcite surface was recognized when calcite dissolved in seawater, resulting in an enriched Mg surface^141^. With the growth of the secondary minerals covering the calcite surface, the dissolution of calcite was inhibited accordingly. Note that almost half of the components of these secondary minerals are liquid sources, which combine with dissolved calcium or carbonate from calcite.

Recently, hydrated Mg-carbonate phases have been identified as the secondary precipitates that form during the dissolution of dolomite and magnesite in pure water^130^. For siderite dissolution, the Raman spectra proved the precipitation of iron oxyhydroxide (goethite or ferrihydrite)^130,142^. When siderite dissolved under extreme conditions, a complex assemblage of Fe^II^–Fe^III^-iron oxides forms, which was dominated by wüstite (FeO) but also contained some iron(III) observed as hematite and possibly magnetite or defective wüstite^143^. Except for the hydroxyl group (OH^−^), which is the water source, the other elements of these secondary minerals came from the parent dolomite and magnesite. Similarly, the secondary minerals in our experiment, i.e., Ca-poor smithsonite, U-rich calcite, and Co-poor aragonite, inherited all the components of the original impurity-bearing calcite. A possible pathway for the formation of Ca-poor smithsonite and U-rich calcite could be expressed as Eqs. (1) and (2). The dissolved Zn/Ca molar ratio of Zn-calcite was much less than that of the solids, suggesting that more Ca^2+^ was released than Zn^2+^ (Fig. S7). This phenomenon was consistent with Eq. (1). In contrast, the dissolved U/Ca molar ratio was higher than that in the solid, indicating that more ${\mathrm{UO}}_{2}^{2+}$ ions were released, which provided the possibility of Eq. (2).

1 $${\text{Ca}}_{{\left( {1 - x} \right)}} {\text{Zn}}_{x} {\text{CO}}_{3\left( s \right)} \left( {{\text{Calcite}}} \right) \rightleftharpoons {\text{Zn}}_{y} {\text{Ca}}_{z} ({\text{CO}}_{3} )_{{\left( {y + z} \right)\left( s \right)}} \left( {{\text{Ca } - \text{ poor }}\;{\text{Smithsonite}}} \right) + \left( {1 - x - z} \right){\text{Ca}}^{2 + }_{{\left( {aq} \right)}} + \left( {x - y} \right){\text{Zn}}^{2 + }_{{\left( {aq} \right)}} + \left( {1 - y - z} \right){\text{CO}}_{3(aq)}^{2 - } \;\;\;\;\;\;\;\left( {x > y,y > z,\frac{y}{z} > \frac{x}{1 - x}} \right)$$

2 $${\text{Ca}}_{{\left( {1 - x} \right)}} ({\text{UO}}_{2} )_{x} {\text{CO}}_{3\left( s \right)} \left( {{\text{Calcite}}} \right) \rightleftharpoons {\text{Ca}}_{y} ({\text{UO}}_{2} )_{z} ({\text{CO}}_{3} )_{{\left( {y + z} \right)\left( s \right)}} \left( {{\text{U } - \text{ rich}}\; {\text{Calcite}}} \right) + \left( {1 - x - y} \right){\text{Ca}}^{2 + }_{{\left( {aq} \right)}} + \left( {x - z} \right)({\text{UO}}_{2} )^{2 + }_{{\left( {aq} \right)}} + \left( {1 - y - z} \right){\text{CO}}_{3 (aq)}^{2 - } \;\;\;\;\; \left( {x > z,y > z,\frac{z}{y} > \frac{x}{1 - x}} \right)$$

As a typical and well known polymorphic transition, the hydrothermal transformation of aragonite to calcite provides a perfect example of interface-coupled dissolution-reprecipitation^144^. The reverse transformation of calcite to aragonite is also relatively common in nature^145^, though it is usually simulated in the laboratory under high-temperature/high-pressure conditions^146–148^. Remarkably, Huang et al. observed the complete transition of high Mg-calcite contents to low Mg-aragonite contents at room temperature/pressure within days^149^. Probably, the formation of Co-poor aragonite in our experiment occurs by a similar mechanism because aragonite is the most stable polymorph of calcium carbonate in the presence of a significant amount of Co^2+^ ions. As mentioned previously, the dissolved Co^2+^ content gradually increased with increasing the dissolution time, and the final released Co^2+^ content observed for Co-08 was as high as 16.13 μM after 2880 h (Fig. 2f). Meanwhile, Co-poor aragonite precipitated as a secondary mineral via the dissolution–precipitation process (Figs. 4e,f, S10a–h). The proposed mechanism is shown in Eq. (3). However, there is no direct evidence at present, thus requiring further study.

3 $${\text{Ca}}_{{\left( {1 - x} \right)}} {\text{Co}}_{x} {\text{CO}}_{{3\left( s \right)}} \left( {{\text{Calcite}}} \right) \rightleftharpoons {\text{Ca}}_{y} {\text{Co}}_{z} ({\text{CO}}_{3} )_{{(y + z)(s)}} \left( {{\text{Co - poor}}\;{\text{Aragonite}}} \right) + \left( {1 - x - y} \right){\text{Ca}}_{{\left( {aq} \right)}}^{{2 + }} + \left( {x - z} \right){\text{Co}}_{{\left( {aq} \right)}}^{{2 + }} + \left( {1 - y - z} \right){\text{CO}}_{{3(aq)}}^{{2 - }} \;\;\;\;\;\left( {x > z,y > z,\frac{z}{y} < \frac{x}{{1 - x}}} \right)$$

### Implication to the environment

Calcite occurs in rocks^1,2^, soils^150^, airborne dust^151,152^, organisms^153^, and even the human body^154,155^ and precipitates as abiogenic and biogenic minerals. In contaminated soils and sediments, bioavailable carbonate-bounded HMs mainly coprecipitate with calcite^156,157^. Moreover, primary carbonate dissolution and secondary carbonate precipitation often happen in soil^158^. Therefore, our results provided further evidence that calcite could control the migration of HMs, especially for Cd, Zn, and Ni. The residual calcite has a higher HM concentration than the primary calcite, which could contribute to the enrichment of Cd and Zn in the soil weathered from limestone^31–34,117^. However, calcite-bound U tends to release during dissolution, suggesting that U might be more sensitive than other metals to soil and ocean acidification. Although the general content of U in natural calcite is 0.1–10 mg/kg and occasionally up to 360 mg/kg^84^, the dissolution of limestone could rein in the uranium concentrations in river water^29^. Meanwhile, the change in the calcite solubility indicates that the stability of a carbon sink in soil and ocean might be reconsidered once HMs were incorporated. Last but not least, the precipitation of smithsonite, Co-poor aragonite, U-rich calcite, and the previously reported rhodochrosite (MnCO_3_) and malachite (Cu_2_(OH)_2_CO_3_) indicated a new mineralization pathway of these minerals, i.e., secondary minerals formed after the dissolution of minerals doped with impurity metals.

## Conclusion

In this study, four divalent heavy metal (Cd^2+^, Co^2+^, Ni^2+^, and Zn^2+^) and uranyl (${\mathrm{UO}}_{2}^{2+}$)-containing calcite were successfully synthesized with changing the M/Ca molar ratio from 0.05 to 12.36%. With the incorporation of impurities, the calcite crystal sizes decreased with increasing the BET surface area. According to the batch dissolution data, the BET surface area calibrated dissolution rates at the early stage decreased with the increase in the impurity molar fraction, indicating an inhibition effect on the dissolution rate. However, both inhibition and promotion effects were observed on the calcite solubility, depending on the type of impurities and their content. The dissolution of impure calcite is incongruent, especially for Cd-, Ni-, and U-containing calcite. The retention ability of Cd and Ni in calcite is remarkably better than that of U, suggesting the potential risk of uranium release due to U-rich calcite dissolution. Meanwhile, the HRTEM and SAED results demonstrated that partial zinc and uranium were immobilized in the reprecipitated phase as smithsonite and U-rich calcite, respectively. Furthermore, the discovery of Co-poor aragonite as a secondary mineral during the dissolution of Co-calcite provided a new type of calcite-aragonite transition. In conclusion, our results demonstrated that the dissolution process of calcite could be significantly changed by incorporating divalent heavy metals and uranyl cations, which has potential environment and climate implications.

## Methods

### Solid preparation and characterization

The coprecipitation method followed the method used by our previous investigation^45^ and was similar to the method used by Katsikopoulos et al.^66^. First, a 0.1 mol/L heavy metal and uranyl solution (M = Cd^2+^, Co^2+^, Ni^2+^, Zn^2+^, ${\mathrm{UO}}_{2}^{2+}$) and a 1 mol/L CaCl_2_ solution were mixed with different molar ratios (Table 1). Afterward, a Na_2_CO_3_ solution was delivered at a constant rate using a peristaltic pump under continuously stirring. The solid-solution precipitates were allowed to age and incubate for three days, and then, the solutions were centrifuged and dried at 80 °C. All the experiments were performed at room temperature and atmospheric pressure. In the following text, the calcite doped with impurities will be recorded as HM-calcite, e.g., Cd-calcite means calcite containing Cd.

The mineral identity of the precipitates was confirmed by X-ray powder diffraction (XRD) using a Bruker D8 Advance powder diffractometer with Ni-filtered Cu–Kα radiation (λ = 1.5406 Å, 40 kV and 40 mA). The samples were scanned from 10° to 80° (2θ), a range that covers the characteristic peaks of calcium carbonate, using a step size of 0.01° (2θ) and a scan rate of 3.0° (2θ) per minute. Then, 0.05 g of each precipitate was dissolved in concentrated HNO_3_ and used to analyze the cation contents, which were determined by inductively coupled plasma-optical emission spectrometry (ICP-OES) (VARIAN VISTA PRO) after dilution. The Brunauer–Emmett–Teller (BET) surface area of the precipitates was determined using a Micromeritics ASAP 2020 M surface area analyzer, in which the samples were dried and degassed at room temperature for 24 h using N_2_ gas. Scanning electron microscopy (SEM) (ZEISS Gemini 500) was performed to obtain images of the precipitates and to provide an approximate size and morphology of the crystalline samples.

### Dissolution experiments and characterization

The procedure of dissolution experiment is the same as described in our previous investigation^45^. In brief, an initial dissolving solution (pH = 5.00 ± 0.05) was prepared using deionized water (18.2 MΩ·cm) and hydrochloric acid (AR). Each 0.125 g of calcite powder was put into a 15 mL polypropylene tube containing 10 mL of the initial reaction solution. Afterward, all the tubes were capped and shaken using an overhead shaker in an incubator chamber at 25 °C. At a specified time, the reaction solution was sampled and filtered by a 0.22 μm pore filter and stored in a clean polyethylene tube. The aqueous pH was measured immediately using a pH meter (Sartorius PB-10). Cations were analyzed using ICP-OES, and the dissolved inorganic carbon (DIC) was measured using a total organic carbon (TOC) analyzer (TOC–VCPH, Shimadzu, Japan). All the dissolution experiments were run in duplicate, and an average was obtained for each metal concentration. Meanwhile, the solids were sampled from the bottles, dried, and preserved for characterization. The morphology of calcite after dissolution was observed by SEM and a high-resolution transmission electron microscopy (HRTEM) system operated at 200 kV (FEI Talos F200S).

### Calculation of solubility and chemical species

The dissolution of pure calcite and impurity-containing calcite can be expressed as Eqs. (4) and (5).

4 $${\mathrm{CaCO}}_{3}\rightleftharpoons {\mathrm{Ca}}^{2+}+{\mathrm{CO}}_{3}^{2-}$$

5 $${\mathrm{Ca}}_{(1-\mathrm{x})}{\mathrm{M}}_{\mathrm{x}}{\mathrm{CO}}_{3}\rightleftharpoons {(1-\mathrm{x})\mathrm{Ca}}^{2+}+{\mathrm{ xM}}^{2+}+{\mathrm{CO}}_{3}^{2-}$$

where $x$ is the molar fraction of MCO_3_ in the solid-solution.

The solubility was calculated using the dissolved metal (Ca + M) and stoichiometric carbonate.

The corresponding equilibrium solubility product expression is showing as Eqs. (6) and (7).

6 $${\mathrm{K}}_{\mathrm{sp}(\mathrm{calcite})}=\left[{\mathrm{Ca}}^{2+}\right]\bullet {[\mathrm{CO}}_{3}^{2-}]$$

7 $${\mathrm{K}}_{\mathrm{sp}(\mathrm{impure calcite})}={\left[{\mathrm{Ca}}^{2+}\right]}^{1-\mathrm{x}}\bullet {\left[{\mathrm{M}}^{2+}\right]}^{\mathrm{x}}\bullet [{\mathrm{CO}}_{3}^{2-}]$$

Usually, the ion activity product (IAP) is also used to describe the solubility of a solid^41^, as shown in Eqs. (8) and (9).

8 $${\mathrm{IAP}}_{(\mathrm{calcite})}={\mathrm{\alpha }}_{{\mathrm{Ca}}^{2+}}\bullet {\mathrm{\alpha }}_{{\mathrm{CO}}_{3}^{2-}}$$

9 $${\text{IAP}}_{{\left( {\text{impure calcite}} \right)}} = \left( {{\upalpha }_{{{\text{Ca}}^{2 + } }} } \right)^{{1 - {\text{x}}}} \cdot \left( {{\upalpha }_{{{\text{M}}^{2 + } }} } \right)^{{\text{x}}} \cdot \left( {{\upalpha }_{{{\text{CO}}_{3}^{2 - } }} } \right)$$

where ${\mathrm{\alpha }}_{i}$ is the activity of species $i$.

However, Davis et al. used the same IAP expression for Mg-calcite that was used for pure calcite^44^.

In this investigation, the species activity and saturation index (Table S1) were calculated using Visual MINTEQ. For comparison, IAP was expressed in the same way as it was expressed by Davis et al.^44^.

## Supplementary information

Supplementary Information.

## Data Availability

The datasets generated during and/or analyzed during the current study, whether they were included in this published article and its Supplementary Information files or not, are available from the corresponding author upon reasonable request.
