# Supplementary material for: Divalent heavy metals and uranyl cations incorporated in calcite change its dissolution process
Source: Sci Rep. 2020 Oct 8;10:16864. doi: 10.1038/s41598-020-73555-6 (PMC7546630; doi:10.1038/s41598-020-73555-6)
Supplement: Supplementary file 1 — Supplementary Information. [file 41598_2020_73555_MOESM1_ESM.docx]

# Supplementary Information

# Divalent Heavy Metals and Uranyl Cations Incorporated in Calcite Change its Dissolution Process

**Xiaohang Zhang^1, 2^, Jianan Guo^1, 2^, Shijun Wu^1,^*, Fanrong Chen^1^, Yongqiang Yang^1^**

**^1^CAS Key Laboratory of Mineralogy and Metallogeny / Guangdong Provincial Key Laboratory of Mineral Physics and Materials, Guangzhou Institute of Geochemistry, Chinese Academy of Sciences, 511 Kehua Street, 510640 Guangzhou, China.^2^University of Chinese Academy of Science, 19 Yuquan Road, 100049 Beijing, China.**

***E-mail: wus@gig.ac.cn**

**Table S1**. Calculated saturation index (SI) of typical minerals at 2880 h using Visual MINTEQ

| **Sample** | **Aragonite** | **Calcite** | **Vaterite** | **MCO_3_** | |
| --- | --- | --- | --- | --- | --- |
| M0 | -0.181 | -0.037 | -0.604 |  |  |
| Cd-02 | -0.67 | -0.526 | -1.092 | Otavite  CdCO_3_ | -0.913 |
| Cd -04 | -0.558 | -0.414 | -0.981 |  | -0.761 |
| Cd -06 | -0.508 | -0.364 | -0.93 |  | -0.683 |
| Cd -08 | -0.407 | -0.263 | -0.829 |  | -0.595 |
| Cd -10 | -0.423 | -0.279 | -0.846 |  | -0.602 |
| Zn-02 | -0.441 | -0.298 | -0.864 | Smithsonite  ZnCO_3_ | -1.393 |
| Zn-04 | -0.34 | -0.196 | -0.763 |  | -1.03 |
| Zn-06 | -0.285 | -0.141 | -0.708 |  | -0.937 |
| Zn-08 | -0.188 | -0.044 | -0.61 |  | -0.856 |
| Zn-10 | -0.054 | 0.09 | -0.476 |  | -0.499 |
| Co-02 | -0.31 | -0.166 | -0.733 | Sphaerocobaltite  CoCO_3_ | 0.665 |
| Co-04 | -0.253 | -0.109 | -0.675 |  | 0.76 |
| Co-06 | -0.142 | 0.002 | -0.565 |  | 0.865 |
| Co-08 | -0.055 | 0.089 | -0.477 |  | 1.102 |
| Ni-0.04 | -0.441 | -0.297 | -0.864 | Gaspéite  NiCO_3_ | -0.091 |
| Ni-0.08 | -0.361 | -0.217 | -0.783 |  | -0.519 |
| Ni-0.2 | -0.459 | -0.315 | -0.881 |  | -0.266 |
| U-0.5 | -0.494 | -0.35 | -0.916 | Rutherfordine  UO_2_CO_3_ | -3.065 |
| U-01 | -0.488 | -0.344 | -0.911 |  | -2.853 |
| U-02 | -0.659 | -0.516 | -1.082 |  | -2.445 |
| U-04 | -0.052 | 0.092 | -0.474 |  | -3.54 |

**Table S2**. The concentration of Ca^2+^ and corresponding M^2+^ during the dissolution of different calcite samples at 2 h and 2880 h

| Sample | Ca^2+^ (mM) | | M^2+^ (μM) | |
| --- | --- | --- | --- | --- |
|  | 2 h | 2880 h | 2 h | 2880 h |
| M0 | 0.16 | 0.52 |  |  |
| Cd-02 | 0.14 | 0.40 | 0.009 | 0.05 |
| Cd-04 | 0.13 | 0.42 | 0.010 | 0.06 |
| Cd-06 | 0.14 | 0.45 | 0.007 | 0.07 |
| Cd-08 | 0.14 | 0.49 | 0.009 | 0.07 |
| Cd-10 | 0.15 | 0.49 | 0.011 | 0.08 |
| Zn-02 | 0.18 | 0.46 | 0.04 | 0.20 |
| Zn-04 | 0.22 | 0.46 | 0.09 | 0.39 |
| Zn-06 | 0.20 | 0.48 | 0.24 | 0.46 |
| Zn-08 | 0.20 | 0.56 | 0.48 | 0.54 |
| Zn-10 | 0.23 | 0.56 | 0.58 | 0.99 |
| Co-02 | 0.13 | 0.55 | 0.60 | 8.15 |
| Co-04 | 0.16 | 0.57 | 0.77 | 9.33 |
| Co-06 | 0.17 | 0.59 | 0.81 | 9.63 |
| Co-08 | 0.22 | 0.69 | 1.23 | 16.13 |
| Ni-0.04 | 0.15 | 0.50 | 0.06 | 0.58 |
| Ni-0.08 | 0.14 | 0.47 | 0.02 | 1.20 |
| Ni-0.2 | 0.07 | 0.33 | 0.04 | 1.29 |
| U-0.5 | 0.15 | 0.45 | 0.76 | 34.78 |
| U-01 | 0.16 | 0.46 | 4.92 | 60.18 |
| U-02 | 0.17 | 0.49 | 22.85 | 70.58 |
| U-04 | 0.31 | 0.58 | 25.24 | 84.76 |

**Table S3**. Linear fitting equations for the solubility of calcite and the molar fraction of metals

| Metal | Equation | R^2^ |
| --- | --- | --- |
| Cd | y=1.1347x+37.927 | 0.9562 |
| Co | y=2.0405x+52.137 | 0.6943 |
| Cu | y=11.98x+64.674 | 0.8579 |
| Mn | y=8.1313x+59.911 | 0.9764 |
| Ni | y=-69.646x+52.43 | 0.9941 |
| U | y=14.021x+50.463 | 0.9783 |
| Zn | y=1.3063x+43.644 | 0.7895 |
| Note: x means the molar fraction of metals, y means the solubility. (The data of Cu and Mn were taken from Minerals 2018, 8, 484) | | |


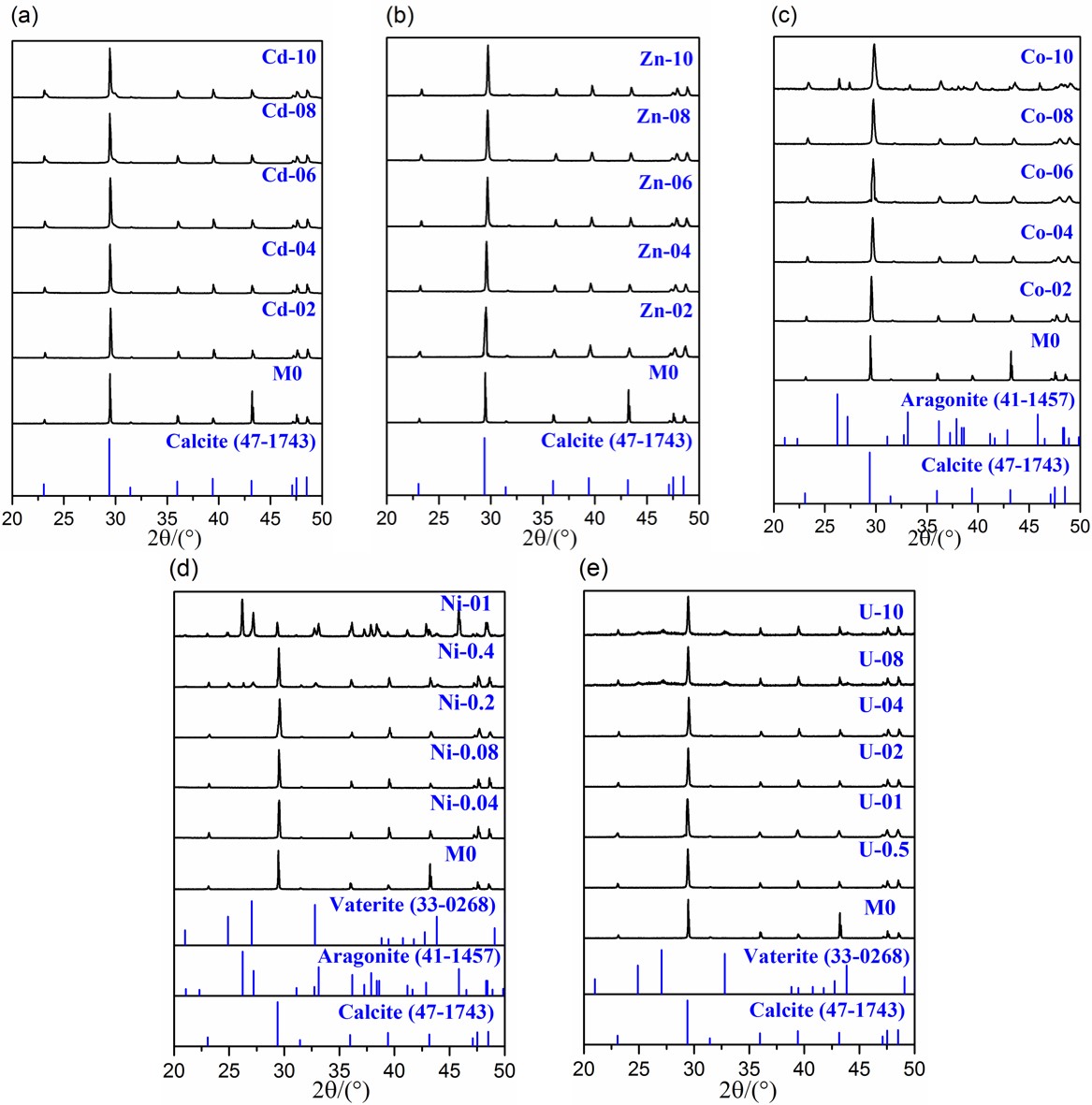
**Figure S1**. Powder XRD patterns of the synthesized products incorporated with Cd (a), Zn (b), Co (c), Ni (d), and U (e).





**Figure S2**. Variation of BET surface area according to the molar fraction of incorporated metals and the corresponding liner simulated equations. The data of Cu and Mn were taken from Zhang et al. (2018).

**
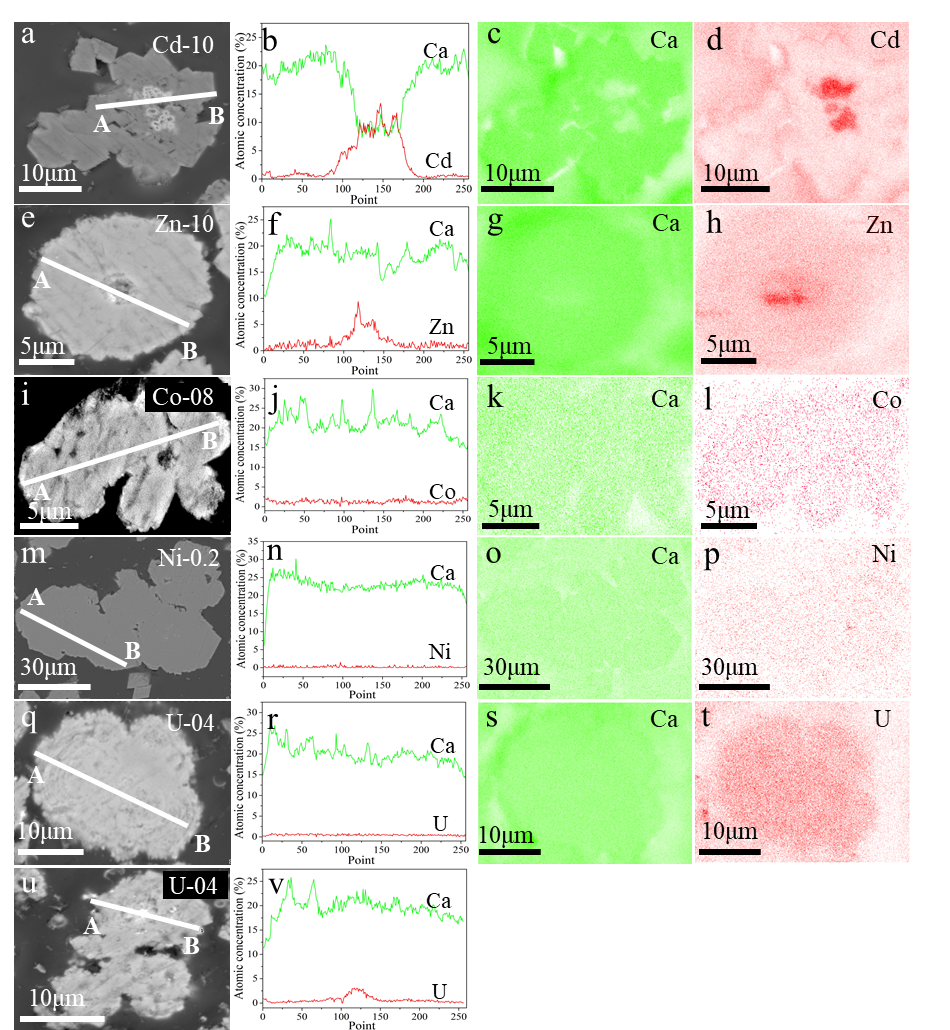
Figure S3.** Spatial distribution of metals in typical polished HM-calcite. The 1^st^ column showed the SEM images. The 2^nd^ column showed the compositional profile corresponding to the EDS line analyses carried out along A–B (250 points per line) in the 1^st^ column. The 3^rd^ and 4^th^ column showed the EDS elemental mapping images of metals (The concentration of metals positively correlated to the brightness).





**Figure S4**. The proportion of dissolved pure calcite after 20 and 120 minutes varied with initial solution pH





**Figure S5**. Evolution of aqueous pH during the dissolution of calcite.

1. Cd-calcite, (b) Zn-calcite, (c) Co-calcite, (d) Ni-calcite, (e) U-calcite





**Figure S6**. The calibrated dissolution rate of calcite within the first two hours. The data of Cu and Mn were taken from Zhang et al. (2018).

**

Figure S7**. The evolution of dissolved HMs/Ca molar ratio during the dissolution of calcite. The dash and dot line indicated the lowest and the highest HMs/Ca molar ratio in solid. (a) The dissolved Cd/Ca molar ratio was 0.04-0.40‰, which was less than the corresponding value of Cd/Ca molar ratio in Cd-calcite. (b) The dissolved Zn/Ca molar ratio was 0.17-2.69‰, which was less than the corresponding value of Zn/Ca molar ratio in Zn-calcite. (c) The dissolved Co/Ca molar ratio increased gradually with the dissolution time. After 2880 h, the Co/Ca molar ratio was less than that of the corresponding solid sample except Co-02. (d) The dissolved Ni/Ca molar ratio at 2880 h was 0.15-4.73‰, which was higher than the corresponding value of Ni/Ca molar ratio in Ni-calcite. (e) The dissolved U/Ca molar ratio at 2880 h was 7.22-18.37%, which was higher than the corresponding value of U/Ca molar ratio in the U-calcite.


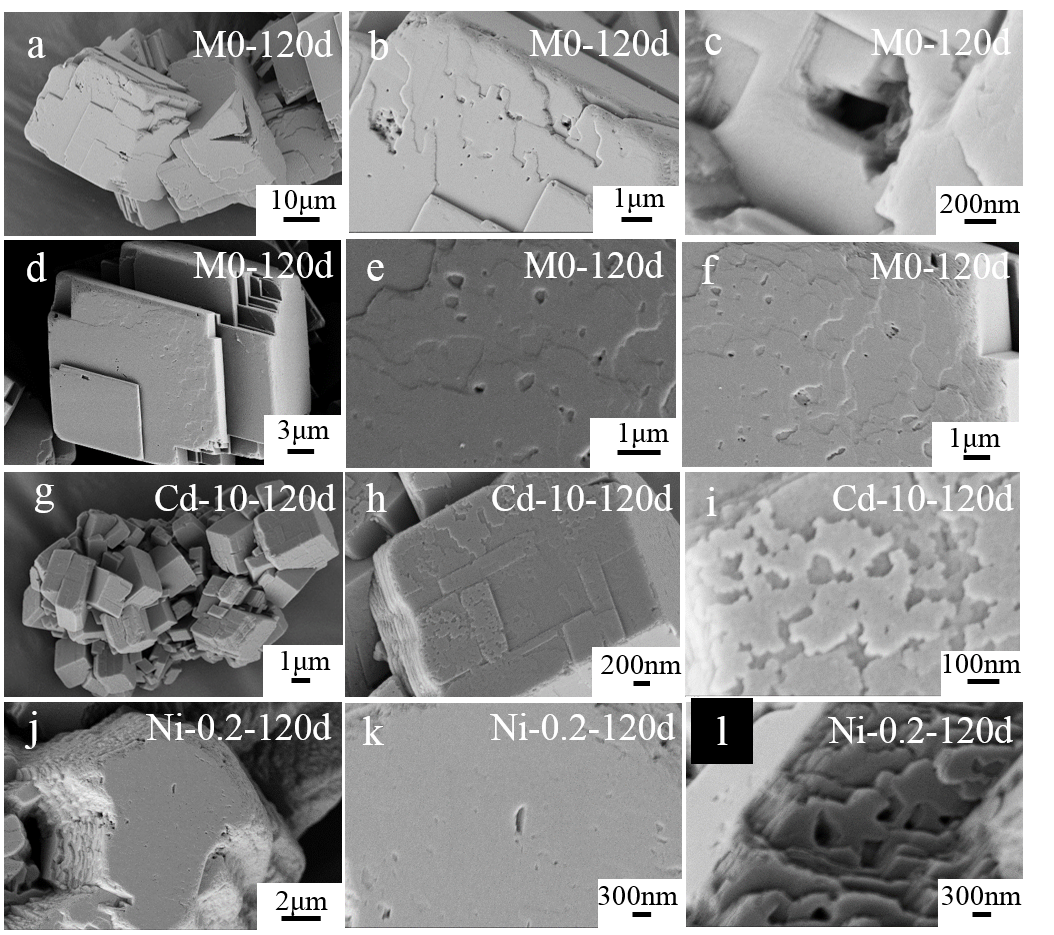


**Figure S8**. SEM images of pure calcite and selected Cd/Ni-calcite after dissolution for 120d. Sample names on the top-right of each figure were labelled as *sample name-T*, e.g., Cd-10-120d stands for Cd-10 after 120 days dissolution.

**
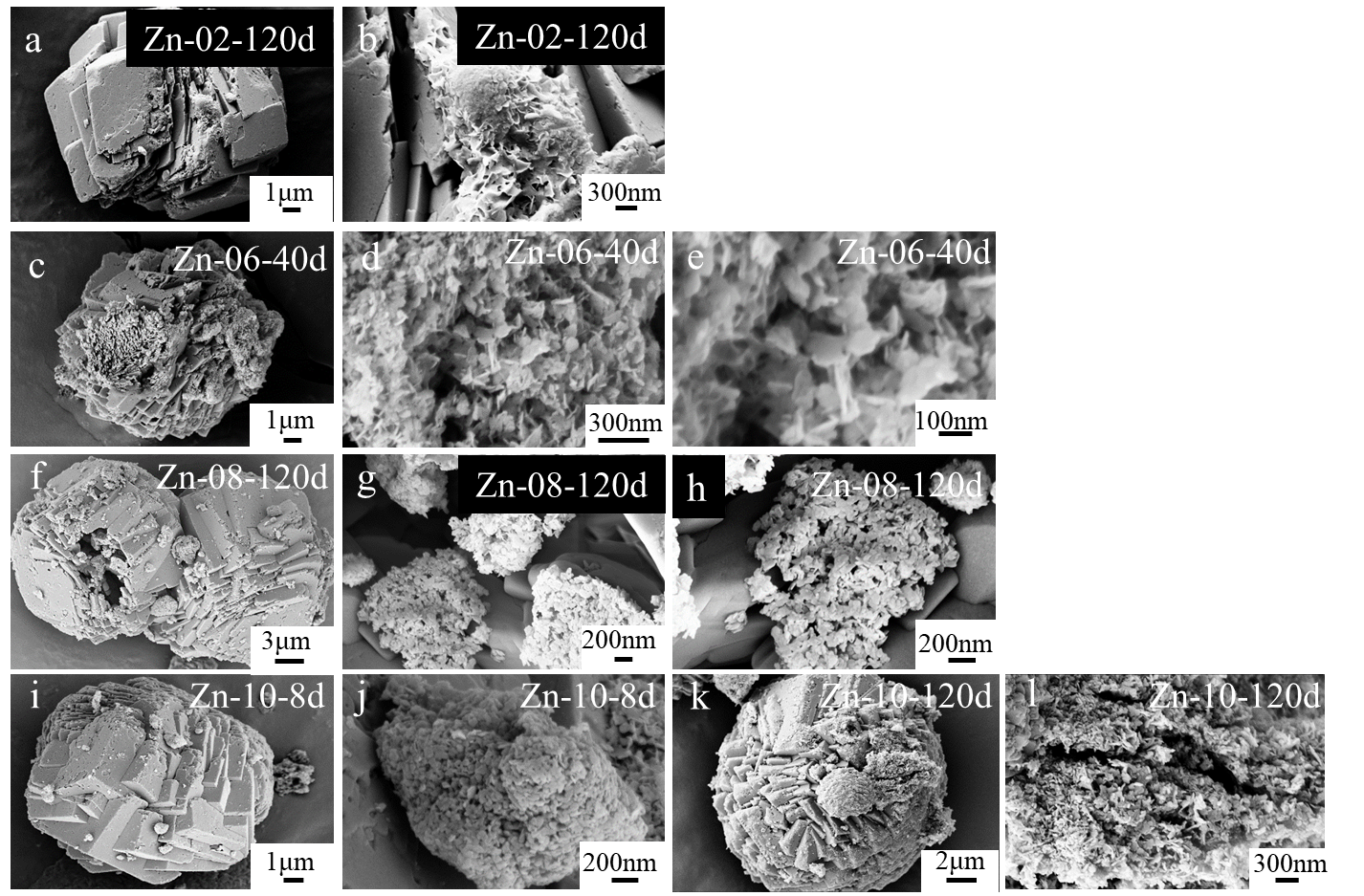
**

**Figure S9**. SEM images of selected Zn-calcite after dissolution. Sample names on the top-right of each figure were labelled as *sample name-T*, e.g., Cd-10-120d stands for Cd-10 after 120 days dissolution.


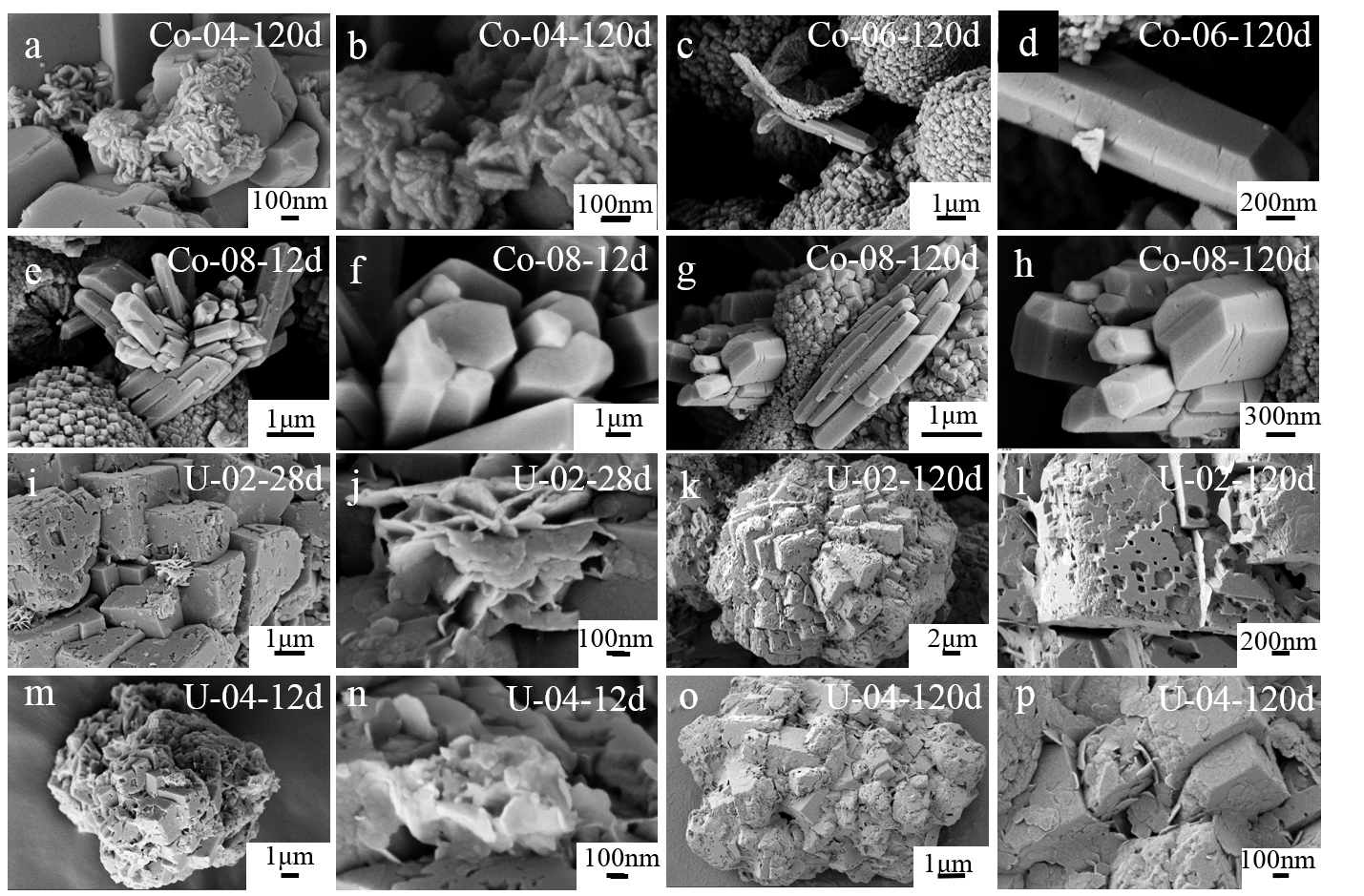


**Figure S10**. SEM images of selected Co/U-calcite after dissolution. Sample names on the top-right of each figure were labelled as *sample name-T*, e.g., Cd-10-120d stands for Cd-10 after 120 days dissolution.


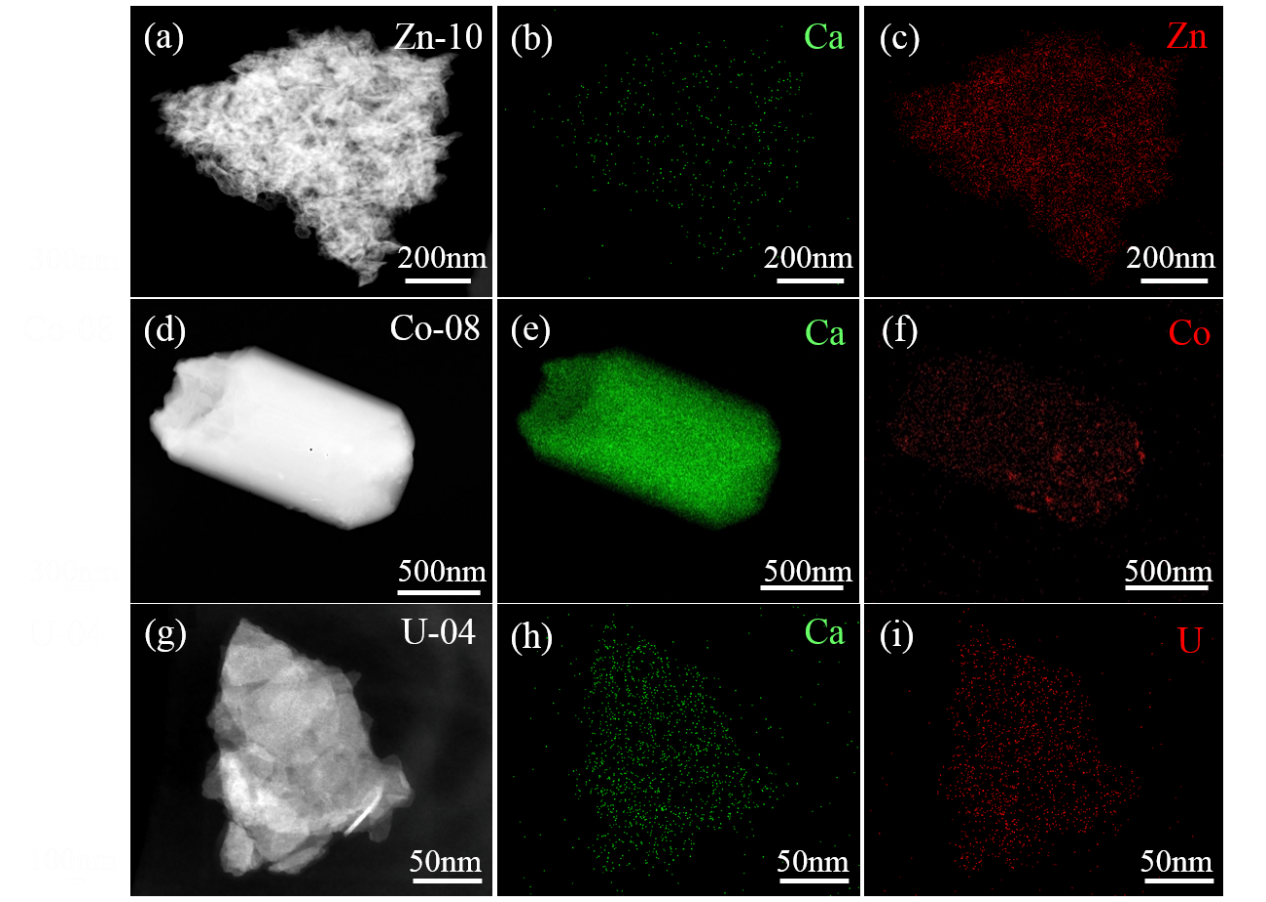


**Figure S11**. TEM images and corresponding EDS elemental mapping images of typical secondary mineral
